# Medication Errors in Secondary Care Hospitals in Kuwait: The Perspectives of Healthcare Professionals

**DOI:** 10.3389/fmed.2021.784315

**Published:** 2021-12-20

**Authors:** Fatemah M. Alsaleh, Sara Alsaeed, Zahra K. Alsairafi, Noor B. Almandil, Abdallah Y. Naser, Tania Bayoud

**Affiliations:** ^1^Department of Pharmacy Practice, Faculty of Pharmacy, Kuwait University, Hawalli, Kuwait; ^2^Department of Clinical Pharmacy Research, Institute for Research and Medical Consultations (IRMC), Imam Abdulrahman Bin Faisal University, Dammam, Saudi Arabia; ^3^Department of Applied Pharmaceutical Sciences and Clinical Pharmacy, Faculty of Pharmacy, Isra University, Amman, Jordan

**Keywords:** Kuwait, medications errors, perception, secondary health care, healthcare professionals

## Abstract

**Objectives:** Medication errors (MEs) are the most common cause of adverse drug events (ADEs) and one of the most encountered patient safety issues in clinical settings. This study aimed to determine the types of MEs in secondary care hospitals in Kuwait and identify their causes. Also, it sought to determine the existing system of error reporting in Kuwait and identify reporting barriers from the perspectives of healthcare professionals (HCPs).

**Material and Methods:** A descriptive cross-sectional study was conducted using a pre-tested self-administered questionnaire. Full-time physicians, pharmacists, and nurses (aged 21 years and older) working in secondary care governmental hospitals in Kuwait were considered eligible to participate in the study. Descriptive statistics and the Statistical Package for Social Science Software (SPSS), version 27 were used to analyze the data.

**Results:** A total of 215 HCPs were approached and asked to take part in the study, of which 208 agreed, giving a response rate of 96.7%. Most HCPs (*n* = 129, 62.0%) reported that the most common type of ME is “prescribing error,” followed by “compliance error” (*n* = 83; 39.9%). Most HCPs thought that a high workload and lack of enough breaks (*n* = 128; 61.5%) were the most common causes of MEs, followed by miscommunication, either among medical staff or between staff and patients, which scored (*n* = 89; 42.8%) and (*n* = 82; 39.4%), respectively. In the past 12 months, 77.4% (*n* = 161) of HCPs reported that they did not fill out any ME incident reports. The lack of feedback (*n* = 65; 31.3%), as well as the length and complexity of the existing incident reporting forms (*n* = 63; 30.3%), were the major barriers against reporting any identified MEs.

**Conclusions:** MEs are common in secondary care hospitals in Kuwait and can be found at many stages of practice. HCPs suggested many strategies to help reduce MEs, including proper communication between HCPs; double-checking every step of the process before administering medications to patients; providing training to keep HCPs up to date on any new treatment guidelines, and computerizing the health system.

## Introduction

At each step of the medication use process (procuring, prescribing, dispensing, administering, and monitoring patients' responses), there is a possibility of an error occurring. Such MEs could affect patient safety, resulting in admission, readmission and/or a longer hospital stay, and can be fatal (1). MEs are the most common cause of adverse drug reactions (ADEs) and one of the most encountered patient safety issues in clinical settings (2). According to the United States National Coordinating Council for Medication Error Reporting and Prevention (US-NCCMERP), MEs are defined as “any preventable events that may cause or lead to inappropriate medication use or patient harm while the medication is within the control of the HCP, patient, or consumer” (3). These events could be related to professional practice, healthcare products, procedures, systems, including prescribing, order communication, product labeling, packaging, compounding, dispensing, distribution, administration, education, or monitoring. In other words, any error independent of its acuity at any point in the medication use system from the time the drug is ordered until the patient receives it is known as an ME (2).

Examples of MEs could include giving a medication to the wrong patient, giving the wrong dose of a medication, not prescribing a medication that was indicated, entering an order for the wrong patient, or forgetting to give a medication that was due. MEs and ADEs are common and can lead to considerable patient harm and increased healthcare costs. Within this context, it has been estimated that 5–10% of all hospitalizations are drug-related, of which 50% of them are avoidable (4). A report from the Institute of Medicine indicated that in the United States (US), at least 1.5 million people get injured, up to 98,000 patients die, and a total of $3.5 billion are spent on treating ME-related injuries every year (5). Worldwide, the cost of MEs has been estimated as $42 billion per year (6, 7).

Identifying the causes and risk factors associated with MEs is crucial to their prevention. A recent systematic review showed that prescribing errors were one of the most common types of ME (comprising up to 94% of MEs; extracted from 46 studies), followed by monitoring errors (up to 73% of MEs; one study), drug-drug interaction errors (up to 58% of MEs; 11 studies), and drug-disease interaction errors (up to 10% of MEs; one study). Considering that MEs are universally under-reported, the incidence rate of MEs is speculated to be even higher. As indicated by the World Health Organization, several patient, HCP, and medication-related risk factors can contribute to MEs. These include poor communication, poor coordination of care, use of medical abbreviations, sound-alike and look-alike drugs, inadequate labeling, staff shortages, and heavy workloads (8). The existing literature indicates that the causes and risk factors of MEs differ across countries and in different settings; hence, investigating them in a specific clinical context in different countries is vital (9).

One of the strategies used to reduce MEs is to encourage their reporting so that suitable solutions can be planned and executed to prevent them from re-occurring. Engaging with colleagues to improve service delivery was also considered effective in reducing potential MEs. In addition, error prevention could be achieved by implementing protocols that would prioritize patient safety, placing it at the center of all caring activities. In Kuwait, the healthcare system is divided into primary, secondary, and tertiary care. Primary care is delivered through general and specialized polyclinics situated across five healthcare regions, secondary care is provided through six general hospitals, and tertiary care is delivered through 15 specialized centers (10). In line with the Ministry of Health's (MOH) vision to improve healthcare quality standards, Accreditation Canada International (ACI) was commissioned in 2008 to introduce a national accreditation program. As part of the certification process, the performance of hospitals needed to meet the national standards of excellence in all aspects of healthcare, from patient safety and ethics, to staff training and education (11). To our knowledge, this is the first study to explore MEs in secondary care hospitals in Kuwait from the perspectives of HCPs, exploring their causes and preventive strategies.

## Materials and Methods

### Study Area and Design

Kuwait is a Middle Eastern country with an area of 17,820 km^2^ and a population of 4,301,359 (2020 estimate) (12). A quantitative, prospective, and cross-sectional study using a self-administered questionnaire was undertaken from February 16th 2020 to February 24th, 2020.

### Study Settings

This study was conducted in four main secondary care hospitals in Kuwait: Al-Jahra, Al-Amiri, Al-Farwaniyah, and Al-Adan. Secondary care hospitals were chosen due to the high number of patients, prescriptions, and workload. Therefore, they have higher ME rates than primary care units.

### Study Participants

The study included English-speaking and full-time working physicians, pharmacists, and nurses, aged 21 years or older. A random selection was made at the level of the hospitals to select four sites for data collection. Then, a proportional number of HCPs were recruited from each hospital based on the total population of physicians, pharmacists, and nurses. To ensure the study's aims and objectives could be best met, certain hospital departments with high medication handling services were selected, including internal medicine, the emergency department, pediatrics, and the pharmacy. The departments with little or no medication handling were excluded from the study, such as the laboratory, and the radiology, dental and surgical units.

### The Questionnaire

The questionnaire was designed based on an extensive literature review related to MEs (Appendix 1) (13–16). The questionnaire was divided into four sections. The first section included five questions covering the types and causes of MEs. The second section comprised six questions seeking to identify errors in the reporting systems in the hospitals, two questions about barriers to reporting and strategies used to improve reporting, and one question regarding strategies to prevent MEs. The third section included 19 (5-point Likert scale) questions to explore the perceptions of HCPs regarding MEs in terms of causes, reporting, and prevention. Of these, eight questions (15–18, 25, 26, 30 and 33) were used to measure HCP's attitudes toward ME reporting, and whether they considered it during their practice. These questions asked the HCPs about the degree of applicability of each question (statement) to their practice using a 5-point Likert scale. The response options ranged from 1 to 5, where 1 meant “strongly disagree” and 5 meant “strongly agree.” Two questions (questions numbered 18 and 25) were negatively worded; thus, they were reverse scored during the analysis, where 1 meant “strongly agree” and 5 meant “strongly disagree.” The total possible score “sub-scale” for the eight questions of section three in the questionnaire that described HCPs' attitudes toward MEs reporting ranged between 5 and 40. The results could be interpreted based on the midpoint of the highest possible score of the scale (equal to 20): the higher the score, the better the attitude toward ME reporting. The final section comprised of 10 questions about demographic information. The HCPs were personally invited to take part in the study; those who agreed were asked to sign a consent form. The participants were also assured that all the information collected would be confidential and anonymized, and that they were free to withdraw at any time.

### Piloting Phase

A pilot study was conducted with different HCPs to ensure the feasibility of the study procedures and confirm whether the questions were clear. The pilot study was carried out in two government hospitals (Al-Amiri and Al-Farwaniyah) and involved 15 HCPs (*n* = 5 physicians, *n* = 5 pharmacists, and *n* = 5 nurses).

### Sample Size

The sample size was calculated using the online Roasoft^R^ calculator based on the assumption that the proportion of responses to most of the main questions would be 50. Using a margin of error of 5% and a Confidence Interval (CI) of 95%, the minimum sample size was determined to be 377. Assuming a response rate of 80%, a larger sample size of 470 was approached. Table 1 shows the total number of HCPs in each recruited hospital.

**Table 1:** Number of HCPs in each hospital, obtained during the preliminary fieldwork.

	**Al-Amiri**	**Al-Farwaniyah**	**Al-Adan**	**Al-Jahra**
Physicians	576	535	656	533
Pharmacists	79	53	100	79
Nurses	1,214	2,124	2,375	1,995
Total	1,869	2,712	3,131	2,607
Number of HCPs to be invited	85	124	142	119

*N.B., Total population in all hospitals = 10,319*.

### Statistical Analysis

Continuous variables were reported as mean [±standard deviation (SD)], while categorical variables were reported as frequencies and percentages. The Kolmogorov–Smirnov and Shapiro Wilk tests were used to check the normality of the data. The participants' scores were interpreted on a continuous scale based on the scale midpoint, where scores above the midpoint represented more positive attitudes toward ME reporting for that factor. The one-way ANOVA test was used to compare the mean scores between different demographic groups. Tukey's honestly significant difference (HSD) *post-hoc* test was conducted to identify the source of significant variation within each group. Multiple linear regression analysis was used to identify predictors of positive attitudes toward ME reporting. A CI of 95% (*p* < 0.05) was applied to represent the statistical significance of the results, and the level of significance was pre-determined as 5%. The Statistical Package for Social Science Software (SPSS), version 27 was used for the data analysis.

## Results

### Demographics and Background Information

A total of 470 HCPs were approached and invited to take a part in the study, of which 255 refused to participate due to their own reasons, not having the time or not being interested in the study topic. Two hundred and fifteen questionnaires were distributed to HCPs working in the four selected hospitals, of which 208 were completed and returned, giving a completeness rate of 96.7%. The median age of the respondents was 33.5 years, with a range of 22–68 years old. The majority of the respondents were female (*n* = 129; 62%) and overseas graduates (*n* = 179; 86.1%), having the minimum of a bachelor's degree in their specialty (*n* = 153; 73.6%). Most of the respondents were nurses (*n* = 125; 60.1%) and Indians (*n* = 102; 49%), and they worked primarily in the general medicine department (*n* = 86; 41%). Around one-third of the respondents had 1 to 5 years (*n* = 61; 29.3%) of experience, with a median of 8 years of practice. Table 2 shows the characteristics of the study participants.

**Table 2:** Demographic characteristics of the respondents.

	**Number (%)**
**Age (years)**	
21–30 31–40 41–50 51–60 >60 **Median age** (min–max)	73 (35.1) 87 (41.8) 35 (16.8) 12 (5.8) 1 (0.5) 33.5 (22–68)
**Gender**	
Male Female	79 (38.0) 129 (62.0)
**Nationality**	
Indian Kuwaiti Egyptian Philippino Jordanian Syrian Bahraini	102 (49.0) 68 (32.7) 20 (9.6) 11 (5.3) 4 (1.9) 2 (1.0) 1 (0.5)
**Education level**	
Diploma Bachelor Master Ph.D Pharm.D	27 (13) 153 (73.6) 19 (9.0) 7 (3.4) 2 (1.0)
**Institution of graduation**	
Kuwait University Overseas Universities	29 (13.9) 179 (86.1)
**Duration of experience (years)**	
<1 1–5 6–10 11–15 16–20 21–25 >25 **Median experience years** (min–max)	23 (11.0) 61 (29.3) 53 (25.5) 32 (15.4) 21 (10.1) 13 (6.3) 5 (2.4) 8 years (0.3–40)
**Name of hospital**	
Al-Farwaniyah Al-Adan Al-Amiri Al-Jahra	57 (27.4) 57 (27.4) 56 (26.9) 38 (18.3)
**Duration of working in the same hospital (years)**	
<1 1–5 6–10 11–15 16–20 >20	35 (16.8) 74 (35.6) 47 (22.6) 27 (13.0) 15 (7.2) 10 (4.8)
**Specialty of the HCP**	
Nurses Physicians Pharmacists	125 (60.1) 67 (32.2) 16 (7.7)
**Primary working department**	
Internal medicine (non-surgical) Pediatrics Pharmacy Surgery Emergency room Many different units Intensive Care Unit (ICU)	85 (40.9) 59 (28.4) 16 (7.7) 15 (7.2) 14 (6.7) 11 (5.3) 8 (3.8)

### Types of Medication Errors

The most common types of MEs reported by the HCPs were “prescribing errors” (*n* = 129; 62.0%), followed by “compliance errors” (*n* = 83; 39.9%). On the other hand, the least commonly reported type of ME was “unauthorized medication errors” (*n* = 8; 3.8%). Table 3 summarizes the types of MEs as mentioned in the questionnaire.

**Table 3:** Types of MEs mentioned in the questionnaire and their frequency.

**Type of medication error**	**Frequency (%)**
Prescribing error Compliance error Improper dose error Monitoring error Omission error Wrong time error Wrong dosage form error Wrong administration technique error Wrong medication preparation error Deteriorated medication error Unauthorized medication error Poor handwriting that leads to dispensing wrong drugs	129 (62.0) 83 (39.9) 50 (24.0) 50 (24.0) 37 (17.8) 37 (17.8) 32 (15.4) 14 (6.7) 11 (5.3) 10 (4.8) 8 (3.8) 1 (0.5)

When they were asked whether they were involved in any MEs during their practice, only 21.2% (*n* = 44) of the HCPs reported that they had committed them, while the majority (*n* = 164; 78.8%) confirmed that they were not involved in any. The most common MEs, as reported by the respondents, were prescribing wrong doses or wrong medications (*n* = 17; 8.1%) (Table 4).

**Table 4:** Reported MEs by the HCPs.

**Medication error**	**Frequency (%)**
Prescribing wrong dose Prescribing wrong medications Dispensing wrong medications Wrong time errors Inappropriate home medications reconciliation Prescribing wrong dosage form Discharge medication reconciliation omission/inappropriate reconciliation Compliance errors Incorrect counseling Transcription and documentation errors Missing value	17 (8.1) 12 (5.7) 3 (1.4) 3 (1.4) 2 (1.0) 2 (1.0) 1 (0.5) 1 (0.5) 1 (0.5) 1 (0.5) 1 (0.5)

Regarding the stage at which MEs occurred and the involved departments, the majority of the reported MEs occurred during the prescribing stage (*n* = 116; 55.8%), followed by the stage of communicating a medication order (*n* = 91; 43%), and then at the documentation stage (*n* = 50; 24%). The most commonly reported MEs had occurred on hospital wards (*n* = 90; 43.3%), followed by emergency rooms (*n* = 85; 40.9%). Table 5 shows the details of the stages at which MEs had occurred.

**Table 5:** Stage of the medication management process, at which MEs mentioned and their frequency.

**Stage**	**Frequency (%)**
Prescribing Communicating an order Documentation Dispensing Administration Labeling Monitoring Preparation/compounding Transcribing Storing Packaging	116 (55.8) 91 (43.8) 50 (24) 37 (17.8) 34 (16.3) 32 (15.4) 21 (10.1) 21 (10.1) 18 (8.7) 16 (7.7) 15 (7.2)

### Causes of MEs

From the perspective of the HCPs, the medication management, operation and workflow systems in place were the largest contributors to MEs (*n* = 89; 42.8%), followed by fellow staff (*n* = 74; 35.6%), the patients (*n* = 32; 15.4%) and the administration (*n* = 12; 5.8%). One (0.5%) participant indicated that the pharmacy was also responsible. Regarding factors that contributed most to MEs, most HCPs (*n* = 128; 61.5%) reported “heavy workload and lack of enough breaks.” This was followed by “miscommunication among medical staff” (*n* = 89; 42.8%) and “miscommunication between HCPs and patients” (*n* = 82; 39.4%). Table 6 shows the details of the causes of MEs as reported by the HCPs.

**Table 6:** Causes of MEs and their frequency.

	**Frequency (%)**
**Causes of MEs**	
Heavy workload and lack of enough breaks Miscommunication between HCPs Miscommunication between patients and HCPs Handover of medication related information (illegible handwriting, look-alike/sound-alike medications) Lack of electronic hospital information systems Lack of teamwork between HCPs Poor knowledge, experience, and training Complexity of medical care Carelessness of HCPs Incorrect electronic data entry Non-adherence to policies and procedures issued by safety and quality control	128 (61.5) 89 (42.8) 82 (39.4) 76 (36.5) 69 (33.2) 47 (22.6) 30 (14.4) 26 (12.5) 17 (8.2) 1 (0.5) 1 (0.5)

### Potential Impact and Consequences of MEs

From the perspective of the HCPs, MEs resulted in “no patient harm” (*n* = 133; 63.9%); however, some HCPs suggested that MEs caused temporary (*n* = 57; 27.5%) or permanent harm (*n* = 11; 5.2%) to patients' health. Table 7 shows the potential harm and consequences of MEs on patients' health as reported by some HCPs.

**Table 7:** The potential impact and consequences of MEs on patients' health.

	**Frequency (%)**
No harm (e.g., only careful watching, no treatment required) Temporary harm (e.g., Intervention/treatment or hospital admission was required) Near death Permanent harm (e.g., patient disability) Patient death Unknown (e.g., the HCP did not follow-up with the patient)	139 (66.8) 44 (21.2) 13 (6.3) 7 (3.3) 4 (1.9) 1 (0.5)

### Reporting of MEs and Barriers to Reporting

Almost all the HCPs (*n* = 205; 98.6%) reported that they had a system in the hospital to report identified MEs, and they confirmed that the reporting system was paper-based, apart from one participant who reported the use of a mobile application for incident reporting. Only three participants (1.4%) thought that there was no ME reporting system available in their hospital. However, less than a quarter of the HCPs reported that they had filled out an incident report in the past 12 months, while the majority (*n* = 161; 77.4%) had not filled out any reports. When they were asked about the barriers that prevented them from reporting MEs, some HCPs (*n* = 65; 31.3%) reported: “lack of feedback after submitting the report,” followed by “length and complexity of the current incident report forms” (*n* = 63; 30.3%), and “fear of punishment” (*n* = 39; 18.8%) as the major factors. Most HCPs (*n* = 129; 62.0%) confirmed that after identifying an ME and filling out an incident report, the most common actions were to inform the supervisor at the hospital, submit a report to higher authorities and committees, and forward a report to quality control. Table 8 details the actions undertaken after completing an incident report.

**Table 8:** Actions undertaken after filling out an incident report.

**Action**	**Frequency (%)**
Informing the supervisor in the hospital Submitting a report to higher authorities and committees Forwarding a report to quality control I don't know/I did not follow up Discussing the error with a HCP to correct it Keeping a report in the incident reporting box Writing a patient complaint Conducting a study survey to avoid the error Carrying out an investigation	37 (17.7) 28 (13.4) 22 (10.5) 20 (9.6) 11 (5.2) 8 (3.8) 1 (0.5) 1 (0.5) 1 (0.5)

### Strategies to Prevent Medication Errors and Improve Incident Reporting

When HCPs were asked to identify strategies to prevent MEs in the future in hospitals in Kuwait, many suggestions were offered. These included proper communication between HCPs (*n* = 40; 19.2%), double-checking every step of the process before medication administration to patients (*n* = 31; 14.9%), training HCPs to keep them up to date on new treatment guidelines (*n* = 27; 12.9%), legible and proper prescription handwriting (*n* = 24; 11.5%), following the 5-right rules of medication administration (e.g., right patient, right drug, right dose, right route, and right time) (*n* = 16; 7.7%), and the need for a computerized system to reduce errors (*n* = 15; 7.2%). Other opinions, such as decreasing the HCPs' workload (*n* = 13; 6.2%) and the need for clinical pharmacists on wards (*n* = 5; 2.4%) to prevent these errors were also reported. When responding to a 5-point Likert scale, most HCPs (*n* = 165; 79.3%) either “strongly agreed” or “agreed” that patients' knowledge about their medications, complying with the standard operating procedures of healthcare authorities (*n* = 159; 76.4%), and the implementation of electronic systems (*n* = 130; 62.5%) would decrease ME rates. With regard to improving incident reporting, 24.5% (*n* = 51) of HCPs reported the need for a proper feedback mechanism after filling out an incident report to be put in place, 21.6% (*n* = 45) agreed on computerizing incident reporting systems and converting them to an online format, and 16.3% (*n* = 34) addressed the need for short, simple, and easily accessible incident report forms. Finally, 10.1% of HCPs (*n* = 21) indicated that they needed training on how to report an ME. Generally, most HCPs, 62.9% (*n* = 131), either strongly agreed or agreed that if there was a national reporting system in place for MEs they would use it. Table 9 shows the strategies to improve incident reporting and prevent MEs in the future, as perceived by HCPs.

**Table 9:** Strategies to prevent MEs and improve incident reporting.

	**Frequency (%)**
**Strategies to improve incident reporting**	
Providing proper feedback for HCPs after filling out an incident report form Computerizing the reporting process/transferring to an online application Making reporting forms short, simple, and easy to access Providing incentives for HCPs for filling out incident reports Training of HCPs on how to report a ME Avoiding punishment and blaming Providing proper communication between workers and higher authorities Arranging committees to analyze, audit, and review the reports	51 (24.5) 45 (21.6) 34 (16.3) 30 (14.4) 21 (10.1) 12 (5.8) 7 (3.4) 3 (1.4)
**Strategies to prevent/reduce MEs**	
Improving communication between HCPs Double-checking every step before medication administration to patients Training of HCPs to keep them up to date on new treatment guidelines Eligible and proper prescription handwriting Following the 5-right rules of medication administration Needing of computerized systems Decreasing workload on HCPs Increasing number of staff Proper documentation Presence of clinical pharmacists on wards	40 (19.4) 31 (15.0) 27 (13.1) 24 (11.7) 16 (7.8) 15 (7.3) 13 (6.3) 9 (4.4) 6 (2.9) 5 (2.4)

### Perspectives of HCPs About MEs

The majority of HCPs (*n* = 172; 82.7%) either “strongly agreed” or “agreed” that the patient's entire medications list should be reviewed more often, and 143 HCPs (68.7%) either “strongly disagreed” or “disagreed” that if an ME occurred and it did not harm the patient, it was not necessary to report it to management. More than half of the HCPs (*n* = 130; 62.5%) either “strongly agreed” or “agreed” that MEs must be discussed with the concerned patient, while nearly half of them (*n* = 110; 52.9%) either “strongly disagreed” or “disagreed” that it was embarrassing to discuss MEs with colleagues. However, 39.4% of HCPs (*n* = 82) either “strongly agreed” or “agreed” that MEs were a real concern in Kuwait and should be addressed appropriately. The average score for the ME reporting sub-scale was 28.7 (SD: 3.9) out of 40, representing 71.8%. The participants' scores ranged between 18 and 39. There was a statistically significant difference in the mean perception score toward ME reporting based on nationality and place of work (*p* < 0.05). Age was negatively associated with having a positive attitude toward ME reporting (*p* < 0.05). On the other hand, Kuwaiti HCPs, females and those with a master's degree had more positive attitudes toward ME reporting (*p* < 0.05) (Tables 10, 11).

**Table 10:** Mean perception scores toward MEs reporting stratified by demographics of the respondents.

**Variable**	**Mean score (SD)**	***P*** **-value**
**Gender**		
Male Female	28.1 (4.3) 29.1 (3.5)	0.081
**Nationality**		
Indian Kuwaiti Egyptian Philippin Jordanian Syrian Bahraini	27.8 (3.2) 30.0 (4.3) 29.3 (3.5) 26.4 (4.3) 29.3 (3.1) 34.5 (4.9) –	<0.001^*^
**Education level**		
Diploma Bachelor Master Ph.D Pharm.D	28.5 (4.1) 30.1 (3.9) 30.1 (3.0) 31.0 (2.8) 28.3 (2.5)	0.314
**Institution of graduation**		
Kuwait University Overseas Universities	29.7 (4.4) 28.5 (3.8)	0.147
**Name of hospital**		
Al-Farwaniyah Al-Adan Al-Amiri Al-Jahra	29.2 (4.3) 28.5 (3.2) 29.5 (3.7) 27.1 (4.1)	0.02^*^
**Duration of experience (years)**		
<1 1–5 6–10 11–15 16–20 >20	29.2 (4.2) 28.9 (4.2) 28.5 (3.5) 28.4 (3.8) 27.2 (3.6) 28.8 (2.5)	0.661
**Primary working department**		
Internal medicine (non-surgical) Pediatrics Pharmacy Surgery Emergency room Many different units ICU	28.3 (4.1) 28.4 (3.6) 30.8 (3.8) 29.0 (4.1) 27.9 (2.8) 29.5 (5.1) 30.3 (2.1)	0.225

* *p < 0.05*.

**Table 11:** Multiple-linear regression predicting attitudes of HCPs toward MEs reporting.

**Variable**	**B**	**SE**	**ß**
**Age**	−0.109	0.053	−0.247^*^
**Gender—**female	1.367	0.597	0.172^*^
**Nationality—**Kuwaiti	1.790	0.779	0.217^*^
**Education level—**Bachelor	−0.019	0.845	−0.002
Master	2.422	1.173	0.181^*^
Ph.D	3.158	1.705	0.147
Pharm.D	0.507	2.993	0.013
**Institution of graduation**—overseas universities	1.103	0.973	0.099
**Working experience (years)**			
1–5	−0.692	1.128	−0.046
6–10	1.205	1.176	0.075
11–15	0.682	1.090	0.046
16–20	0.721	1.145	0.045
>20	−0.197	1.409	−0.010
**Primary working department**			
Pediatrics	−0.729	1.217	−0.093
Pharmacy	0.124	1.513	0.008
Surgery	−1.193	1.259	–.0139
Emergency room	−0.078	1.571	−0.005
Many different units	1.559	1.787	0.078
ICU	0.885	1.588	0.061
**Constant**	29.554	2.209	
**Adjusted R** ^**2**^			0.076
***P*** **-value**			0.019

* *p < 0.05.*

*B, the average change in the dependent variable associated with a one unit change in the independent variable, statistically controlling for the other independent variables; SE, the standard deviation of its sampling distribution or an estimate of that standard deviation; ß, a statistical measure that compares the strength of the effect of each individual independent variable to the dependent variable*.

## Discussion

To our knowledge, this study is the first one to identify types, causes, and prevention strategies regarding MEs in secondary care hospitals in Kuwait from the perspectives of HCPs. A similar study was conducted in one tertiary hospital in Kuwait by Ahmed et al. (16). Consistent with Ahmed et al.'s study (16), most of the participants in the current study were female (62%) and had a bachelor's degree in their specialty (73.6%). Unsurprisingly, this is because females working in the health sector outnumber males among both the Kuwaiti and non-Kuwaiti population: 61.3 vs. 38.7%, respectively (17).

In the current study, the most common type of ME was prescribing error (62%), which commonly occurred at the prescribing stage, as reported by half of the HCPs (55.8%). Findings from Saudi Arabia and Bahrain the same, with prescribing error scoring 72 and 88%, respectively (18, 19). On the other hand, results from South Korea showed that only 23.3% of the participants reported prescribing error as the most common type of ME (20). High prescribing error rates could be due to illegible handwriting, the use of abbreviations in prescriptions, and a lack of knowledge of updates (e.g., using outdated therapeutic guidelines) (19). One reason behind the non-adherence of HCPs to prescribing guidelines could be due to the lack of up-to-date international guidelines (16, 21). Consistent with Ahmed et al.'s findings (16), this study found that MEs mostly occurred on wards (43.3%) and emergency rooms (40.9%). This could be due to the high drug handling services and workloads in those two departments.

Regarding the causes of MEs, the current study reported that high workloads and a lack of enough breaks (61.5%) were the most common causes of MEs, followed by miscommunication, either among medical staff (42.8%) or between staff and patients (39.4%). Heavy workloads and tiredness have been mentioned as the main causes of MEs in many studies (22). Studies in Turkey, Nigeria, Jordan and South Korea have shown that the most common cause of MEs is high workloads, which scored 68.1%, 78, 41.4, and 40.9%, respectively (20, 23–25). Likewise, Ahmed et al. (16) reported miscommunication either among medical staff (35.4%) or between staff and patients (62.7%) as a common cause of MEs in Kuwait. In the United Kingdom (UK), physicians and nurses working in an intensive care unit (ICU) reported poor written or verbal communication as a risk factor (26). Accordingly, HCPs need to have good communication skills to assist with and prevent MEs. Effective communication skills are crucial to improving communication between HCPs and patients, as well as between collaborating HCPs; this should reduce MEs (27). Other causes of MEs that were less commonly addressed in the current study, such as illegible doctors' handwriting (36.5%), lack of knowledge, experience, and training (14.4%), and the carelessness of HCPs (8.2%), have also been reported in different countries, such as Saudi Arabia, Jordan and Malta (22, 28–30). In the UK, it has also been reported that poor knowledge, experience, and training were common individual factors that contributed to errors. In that study, it was also found that nurses did not always check the prepared doses or a patient's identity before administering medication (26).

The majority of HCPs (78.8%) in this study indicated that they had not committed MEs during their years of practice. Similar responses have been reported by 80.6% of HCPs in Turkey (31). The reasons behind such a response may be due to a perceived fear of disciplinary action, loss of reputation, and, consequently, loss of job. In contrast, 44.6% of HCPs reported that they had encountered MEs during their practice in a tertiary hospital (16). Most of the committed MEs in the current study resulted in no patient harm (63.9%) and, in most cases (62.5%), the required intervention was carefully observed without the need for any intervention. Hospital admissions, as a result of an ME, were required in 13% of cases in this study. It has been estimated that hospital re-admissions and hospitalizations due to MEs in the European Union are between 8 and 12% of all reported cases (32).

Regarding the practice of incident reporting, this study showed that 77.4% of HCPs had not filled out any incident reports in the past 12 months, with only 17.3% had filled out one or two reports. This result is consistent with a study in Qatar, where 66.8% of HCPs had not filled out reports, and 11.7% had filled out one to two reports in the previous 12 months (33). A low level of incident reporting has also been found in Palestinian hospitals, where only 40.3% of MEs have been reported in the past 12 months (34). Comparable results have also been found in Australia, where more than 40% of physicians had never completed an incident report (35). In the USA, as well, around 35% of physicians and nurses declared that they had reported <20% of their perceived MEs and <40% of other staff errors in the past 12 months (36). From the perspective of the HCPs in the current study, the lack of feedback after submitting the report (31.3%), as well as the length and complexity of incident report forms (30.3%), discouraged them from reporting. Similarly, in 2019, in Kuwait, 38.1% of HCPs found that the incident report forms were complex (16). In Saudi Arabia, Australia and the UK, the lack of feedback on previous MEs and the complexity of the reporting system have also been identified by physicians and nurses as leading causes of not reporting MEs (26, 30, 35). In Korea, only 33.5% of pharmacists mentioned that they had received feedback about their errors (1). From the perspective of those pharmacists, the lack of feedback (63.1%), unclear ME reporting protocols (50.3%), heavy workloads (47.7%), lack of harm to the patient (45.7%), and the belief that there was no need to report (43.1%) were the most common reasons for ME under-reporting.

From the perspective of physicians and nurses in the USA, the most common ME reporting barriers included the uncertainty concerning what to report and who should report (40.7%), and the possibility of affecting the careers of fellow HCPs (37%) (36). Similarly, only 42.1% of committed MEs in Jordan and 40.3% in Palestine were reported. The lack of reporting was due to confusion regarding the definition of an ME, when errors should be reported, and the fear of disciplinary action (28, 34). Therefore, raising awareness about the reporting system and including training activities on how to access, complete, and submit an incident form were key initiatives to motivate HCPs to report. The perceived fear of blame or punishment has been mentioned by 18.8% of HCPs in the current study as a barrier for not reporting. This barrier has also been identified by HCPs in other countries, such as Saudi Arabia, UK, Malta and Korea (22, 29, 30, 37). In Taiwan, nurses reported only 19% of MEs due to self-recrimination and fear of blame (38). In South Korea, only 10% of nurses reported incidents due to worrying about penalties and poor performance evaluations (37).

However, the current study showed a statistically significant difference in the mean perception score toward ME reporting based on nationality and place of work. It has been found that Kuwaiti, Syrian, and Filipino HCPs and those who worked at Al-Farwaniah and Al-Jahra hospitals, had better attitudes toward incident reporting than other HCPs. Age was negatively associated with having a positive attitude toward reporting, whereby younger HCPs reported better attitudes toward incident reporting than older HCPs. This could be due to a perceived fear of shame by older HCPs, who may have felt that they were experienced enough not to commit MEs, and, if they did commit them, they preferred not to report them to avoid punitive consequences and prevent loss of reputation.

Finally, the HCPs in the current study highlighted different strategies that could help improve incident reporting, such as providing continuous feedback on reported errors (24.5%), computerizing the reporting system (21.6%), creating easily accessible and user-friendly reporting forms (16.3%), and avoiding negative blaming attitudes (5.8%). Similarly, Taylor et al. (39) reported potential interventions to improve error reporting from the perspective of physicians and nurses such as providing regular feedback on reported errors (65.4%) and an electronic format for errors reporting (44.9%). In addition, HCPs in the UK pointed out the need to receive information on previously reported MEs, being provided with an anonymous copy of the reported incident, and a move away from a “culture of blame” and to a “culture of medicine” to encourage errors reporting (26, 40).

### Implications for Practice

MEs are problematic and pose a health safety concern in Kuwait and globally. Findings from the current study have highlighted different types of MEs, those encountered most at multiple hospitals in Kuwait. In this regard, the MOH should take into consideration the proposed solutions to overcome these errors and minimize their occurrence. For example, to address administration errors, one of the most perceived types of ME, Pape et al. described a method to reduce their incidence (41). In that study, a group of nurses wore a vest that stated “Do Not Disturb During Medication Administration.” This method has been found to decrease distractions incrementally and, consequently, increase the nurses' focus and time spent on patient care. As a result, a decrease in the incidence of administration related MEs has been observed. In addition, to address and overcome the incidence of MEs due to high workloads and the lack of manpower and enough shift breaks, adequate staffing and a “manpower to workload demand” review is crucial. This could lead to more staff being hired and retained to overcome the distractions which increase the incidence of MEs (28).

This study has presented many barriers and various enabler strategies that could be considered to improve reporting and reduce future errors. Although reporting systems are available to allow feedback on errors in most organizations, these are not always used to their full potential. In this regard, the MOH should apply a standardized, unambiguous protocol for reporting MEs at any stage in the medication use process; this will facilitate the process and improve reporting rates. In addition, feedback on previous MEs should be made available and provided to all staff in a supportive and non-punitive manner. The MOH could also provide motivational training about incident reporting to targeted populations who have poor attitudes toward reporting MEs (e.g., older HCPs and HCPs working in hospitals other than Al-Farwaniyah and Al-Jahra).

### Suggestions for Future Work

As there is a scarcity of studies in Kuwait investigating the types and causes of MEs and the way these errors are being reported, it would be interesting to conduct more studies on a larger population sample. In addition, research evaluating the cost of interventions and hospitalization as a result of MEs should be conducted. This is important to help in predicting the risk and impact of MEs on patients, the government, and the healthcare system.

### Strengths and Limitations

This is the first study that has investigated the types, causes and impact of MEs across all the governorates in Kuwait. It has addressed the situation regarding incident reporting and explored the barriers to reporting, taking into account the HCP characteristics that are associated with better attitudes toward incident reporting. The completeness rate in this study was 96.7%; this may enhance the generalizability of the findings. Moreover, randomizing the hospitals for site selection is another strength. Finally, the study tool was designed, piloted, and assessed on content and face validity. However, there are certain limitations. First, the data collection process was interrupted and terminated due to the severe acute respiratory syndrome coronavirus 2 (SARS-CoV-2) pandemic. Data collection became impossible because of the countrywide pandemic lockdown and the medical professionals being overloaded. Moreover, filling the questionnaire during busy working days was challenging due to high traffic at the hospitals. Finally, as the aim of this study was to determine the existing system of error reporting in Kuwait and identify reporting barriers from the perspectives of healthcare professionals, we did not explore the inter-professional variations between them. We were unable to get the required sample size; therefore, as this might affect their generalizability, our findings should be interpreted carefully, Despite its limitations, this study has uncovered significant ME types, their causes, the reporting barriers and enabling strategies, and has highlighted the need for prevention strategies in secondary care hospitals in Kuwait.

## Conclusion

In conclusion, this study has identified the main perceived types and causes of MEs, the existing systems for reporting errors, the barriers to reporting, and the strategies needed to prevent MEs and improve reporting from the perspective of the HCPs. MEs are common in secondary care hospitals in Kuwait and have occurred at different stages of practice. HCPs have suggested many strategies to help reduce MEs, mainly concerning proper communication between HCPs, double-checking every step of the process before administering medications to patients, providing training to keep HCPs up to date on any new treatment guidelines, and the computerization of the healthcare system. In addition, from the perspective of the HCPs, giving proper feedback after filling out an incident report, computerizing the incident reporting process, and making incident report forms easy to complete, short, and explicit, are effective strategies to encourage HCPs to report identified MEs. Therefore, informing policymakers about such strategies is crucial to reducing future ME occurrences and any potential resulting patient harm, and, thus, improving the quality of healthcare systems in hospitals in Kuwait and ensuring safe medication practices.

## Data Availability Statement

The original contributions presented in the study are included in the article/Supplementary Materials, further inquiries can be directed to the corresponding author.

## Ethics Statement

Ethical approval was obtained from the Standing Committee for Coordination of Health and Medical Research, MOH, and the Health Science Centre Ethics Committee for Student Research, Kuwait University. Additionally, approvals from head directors of the participating hospitals and the directors of the governorates were sought and obtained before distributing the questionnaires.

## Author Contributions

FA created the presented idea, developed the theory, and performed the computations. FA, SA, ZA, NA, AN, and TB contributed to the design and implementation of the research and conducted the literature review and wrote the first draft of the manuscript. SA collected data under the supervision of FA and ZA. AN performed the analytic calculations and performed the numerical simulations. FA and ZA supervised the project. All authors discussed the results and contributed to the final version of the manuscript.

## Conflict of Interest

The authors declare that the research was conducted in the absence of any commercial or financial relationships that could be construed as a potential conflict of interest.

## Publisher's Note

All claims expressed in this article are solely those of the authors and do not necessarily represent those of their affiliated organizations, or those of the publisher, the editors and the reviewers. Any product that may be evaluated in this article, or claim that may be made by its manufacturer, is not guaranteed or endorsed by the publisher.
