# Supplementary material for: Medication Errors in Secondary Care Hospitals in Kuwait: The Perspectives of Healthcare Professionals
Source: Front Med (Lausanne). 2021 Dec 20;8:784315. doi: 10.3389/fmed.2021.784315 (PMC8720773; doi:10.3389/fmed.2021.784315)
Supplement: Supplementary file 1 [file Data_Sheet_1.pdf]

Appendix 1: The Questionnaire

Hospital name/No.:…………….   HCP (specialty)/No.:………………

Medication Errors in Secondary Care Hospitals in Kuwait- Perspectives of Healthcare Professionals

Instructions

This survey asks for your opinions about medication errors, error prevention and reporting in your hospital and will take about 10 to 15 minutes to complete.

- ***"Medication Errors"*** "any **preventable event** that may cause or lead to **inappropriate medication use or patient harm** while the medication is in the control of the healthcare professional or patient."
- In this survey, think of your ***"department"*** as the unit or clinical area of the hospital where you spend *most* of your work time or provide *most* of your clinical services.

SECTION A: TYPES AND CAUSES OF MEDICATION ERRORS

1. What is the most common form of medication error that you encounter during your practice? YOU CAN SELECT MORE THAN ONE ANSWER.

☐a. Prescribing error	e.g. over/under dose, wrong medication/dose, interactions, allergies.
☐b. Omission error	i.e. failure to administer a dose to a patient at scheduled time.
☐c. Wrong time error	i.e. administration of a medication outside scheduled time interval.
☐d. Unauthorized medication error	i.e. administration of a medication not authorized by a legitimate prescriber.
☐e. Improper dose error	i.e. administration of a dose that is greater/less than the prescribed amount.
☐f. Wrong dosage-form error	i.e. administration of a medication in a different dosage form than the prescribed one.
☐g. Wrong medication-preparation error	i.e. a medication incorrectly formulated or manipulated before administration
☐h. Wrong administration technique error	i.e. inappropriate technique in the administration of a mediation.
☐i. Deteriorated medication error	i.e. administration of a medication that has expired.
☐j. Monitoring error	i.e. failure to review a prescribed medication for appropriateness/problems, or failure to use appropriate laboratory data for assessing patient response to a medication.
☐k. Compliance error	i.e. inappropriate patient behaviour regarding adherence to a prescribed regimen.
☐l. Other errors (please specify)	

2. In your opinion, where do most of the medication errors occur? Select ONE answer

- ☐a. Emergency room
- ☐b. Wards
- ☐c. ICU (any type)
- ☐d. Pharmacy
- ☐e. Other (Please specify)

3. In your opinion, at what stage of the healthcare process where most of the medication errors do occur? YOU CAN SELECT MORE THAN ONE ANSWER.

- ☐a. Prescribing
- ☐b. Transcribing
- ☐c. Communicating order
- ☐d. Administering to patient
- ☐e. Monitoring
- ☐f. Documenting
- ☐g. Preparing/compounding
- ☐h. Packaging
- ☐i. Labeling
- ☐j. Dispensing
- ☐k. Storing
- ☐l. Other (Please specify)

4. Who do you think is to blame for most of the medication errors in your department of service? Select ONE answer

- ☐a. Fellow staff
- ☐b. Administration
- ☐c. Patients
- ☐d. System running the facility
- ☐e. Other (Please specify)

5. What is/are the most common cause(s) of medication errors in your department/hospital? YOU CAN SELECT MORE THAN ONE ANSWER.

- ☐a. Miscommunication between patients and medical staff
- ☐b. Miscommunication between medical staff
- ☐c. Lack of teamwork between medical staff
- ☐d. High workload and lack of enough breaks
- ☐e. Poor knowledge, experience and training
- ☐f. Careless medical staff
- ☐h. Complexity of medical care
- ☐i. Lack of computerized system in the hospital
- ☐j. Handover of medication related information (illegible handwriting, look-alike/sound-alike medications)
- ☐k. Other (Please specify)

SECTION B: MEDICATION ERRORS REPORTING, BARRIERS AND PREVENTION

6. Based on your own experience, have you done/committed any "medication errors" during your practice in this hospital?

☐a Yes ☐b. No

If "YES" can you list them (starting from the most common)?

7. What was the most common impact you have had of these medication errors on the patient? Select ONE answer

☐a. Patient death
☐b. Near death (i.e. life-threatening error such as anaphylaxis)
☐c. Permanent harm
☐d. Temporary harm (required treatment/intervention of error, initial or prolonged hospitalization)
☐e. No harm
☐f. Other (Please specify)

8. What was the worst impact you have had resulting from these medication errors? Select ONE answer

☐a. Patient death
☐b. Patient admission to hospital
☐c. Intervention/treatment, but no hospital admission required.
☐d. Careful watching, but no treatment required
☐e. Other (Please specify)

9. Do you have a system in your hospital to report medication errors (i.e. incident reporting)?

☐a. Yes

☐b. No

If "YES", what do you do with these reports?

10. What method do you use in your hospital to report a medication error? Select ONE answer

☐a. Paper
☐b. Website
☐d. Mobile application
☐e. Other (please specify)

11. In the past 12 months, how many incident reports have you filled out and submitted?

☐a. None
☐b. 1 to 2 incident reports
☐c. 3 to 5 incident reports
☐d. 6 to 10 incident reports
☐e. 11 to 20 incident reports
☐f. 21 or more incident reports

12. What are the barriers that you think would prevent healthcare professionals from reporting medication errors (incident reports) in your hospital?

☐a. Incident report forms are too long and complicated
☐b. No feedback is given after submitting the report
☐c. Not important to report if patient is not harmed
☐d. Feel of shame
☐e. Fear of punitive consequences
☐f. Other (please specify)

13. In your opinion, what can be done to improve incident reporting in your hospital?

14. In your opinion, what are the most important actions to reduce or prevent medication errors?

SECTION C: YOUR PERCEPTION

This section describes your perception about medication errors, causes, reporting and prevention.	**Strongly Disagree** ▼	**Disagree** ▼	**Neither** ▼	**Agree** ▼	**Strongly Agree** ▼
**15.** Medication errors are a severe problem in Kuwait	☐1	☐2	☐3	☐4	☐5
**16.** Patient's entire medications should be reviewed more often than is done today in this hospital………………………………………………	☐1	☐2	☐3	☐4	☐5
**17.** Medication errors have to be discussed with the patient in a question.....	☐1	☐2	☐3	☐4	☐5
**18.** It is embarrassing to discuss medication errors with colleagues	☐1	☐2	☐3	☐4	☐5
**19.** Typically, medication error is a result of an error made by individual professional……………………………………………………………...	☐1	☐2	☐3	☐4	☐5
**20.** Insufficient standard operating procedure are often the underlying reason for medication errors…………………………………………...	☐1	☐2	☐3	☐4	☐5
**21.** Long working hours increase the risk of medication errors	☐1	☐2	☐3	☐4	☐5
**22.** A heavy workload increases the number of medication errors................	☐1	☐2	☐3	☐4	☐5
**23.** Over-the-counter drugs used by patients increases the risk of medication errors..	☐1	☐2	☐3	☐4	☐5
**24.** The working environment (e.g. work premise, noise, equipment) influences the number of medication errors………………………………	☐1	☐2	☐3	☐4	☐5
**25.** If a medication error occurs and it does not harm the patient, it is not necessary to report it to the managers…………………………...….	☐1	☐2	☐3	☐4	☐5
**26.** If there was a national anonymous reporting system for medication errors, I would use it	☐1	☐2	☐3	☐4	☐5
**27.** Seamless information flow between different departments (units) in the hospital decreases the number of medication errors…….…………	☐1	☐2	☐3	☐4	☐5
**28.** Changing working routines based on the observed errors and near-misses are the best ways to decrease the number of medication error	☐1	☐2	☐3	☐4	☐5
**29.** Patients' knowledge about medications decreases the number of medication errors……………………………………………………………	☐1	☐2	☐3	☐4	☐5
**30.** Internal reporting of medication errors prevents new medication errors to occur…………………………………………………………………	☐1	☐2	☐3	☐4	☐5
**31.** I have all the necessary tools to prevent medication errors occurring in my practice………………………………………………………………	☐1	☐2	☐3	☐4	☐5
**32.** Implementation of electronic systems (e.g. e-prescribing and e-dispensing/robotics) have decreased the number of medication errors.	☐1	☐2	☐3	☐4	☐5
**33.** It is possible to decrease the number of medication errors by complying with the standard operating procedures of authorities……	☐1	☐2	☐3	☐4	☐5

<u>SECTION D: BACKGROUND INFORMATION</u> - *This information will help in the analysis of the survey results.*

1. **Age ………………..Years**

2. **Gender** ☐a. Male ☐b. Female

3. **Nationality** ☐a. Kuwaiti ☐b. Non-Kuwaiti (please specify)……………..

4. **What is your <u>LAST</u> degree in your speciality?**

☐a. Bachelor ☐c. Master ☐d. PhD ☐e. Other ………………………

5. **Country of graduation (for the Bachelor degree)?** ☐a. Kuwait University ☐b. Overseas university

6. **Total practice years ……………………………………………**

7. What hospital do you work in?

☐ a. Amiri Hospital ☐ b. Farwaniyah Hospital ☐ c. Jahra Hospital ☐ d. Adan Hospital

☐ e. Mubarak Al-Kabeer Hospital ☐ f. Sabah general hospital ☐ g. Jaber Al-Ahmad Hospital

8. How long have you worked in this <u>hospital</u>?

☐a. Less than 1 year
☐b. 1 to 5 years
☐c. 6 to 10 years
☐d. 11 to 15 years
☐e. 16 to 20 years
☐f. 21 years or more

9. **What is your staff position in this hospital? Select ONE answer that best describes your staff position.**

<u>If PHYSICIAN:</u> *(select where appropriate)*

☐a. Consultant
☐b. Senior Specialist
☐c. Specialist
☐d. Senior Registrar
☐e. Registrar
☐f. Assistant Registrar
☐g. General Practitioner
☐h. Resident
☐i. Trainee
☐j. Other (please specify)……………………………

<u>If PHARMACIST:</u> *(select where appropriate)*

☐ a. Beginner Pharmacist (صيدلي مبتدئ)
☐ b. Senior Pharmacist (صيدلي أول)
☐ c. Senior Pharmacy Specialist (اختصاصي أول)
☐ d. Pharmacist (صيدلي)
☐ e. Pharmacy Specialist (اختصاصي صيدلة)
☐ f. Head of Pharmacy Specialist (رئيس اختصاصيين)

<u>If NURSE:</u> ***(select where appropriate)***

- ☐a. Head of Nursing Department
- ☐b. Assistant Head of Nursing Department
- ☐c. Head Nurse of Clinical Training
- ☐d. Assistant Nurse of Clinical Training
- ☐e. Head of Ward
- ☐f. Nurse
- ☐g. Nurse Assistant
- ☐h. Other (please specify)……………………

10. What is your primary work area or unit in this hospital? Select ONE answer.

- ☐ a. Many different hospital units/No specific unit
- ☐ b. Medicine (non-surgical)
- ☐ c. Surgery
- ☐ d. Obstetrics
- ☐ e. Paediatrics
- ☐ f. Emergency room
- ☐ g. ICU (any type)
- ☐ h. Psychiatry/mental health
- ☐ i. Pharmacy
- ☐ j. Anaesthesiology
- ☐ k. Other (please specify)……………………………………………

THANK YOU FOR COMPLETING THIS SURVEY
